# Longitudinal Development of Refractive Error in Children Treated With Intravitreal Bevacizumab or Laser for Retinopathy of Prematurity

**DOI:** 10.1167/tvst.10.4.14

**Published:** 2021-04-15

**Authors:** Michael Simmons, Jingyun Wang, Joel N. Leffler, Shanshan Li, Sarah E. Morale, Angie de la Cruz, Eileen E. Birch

**Affiliations:** 1 Department of Ophthalmology, University of Texas Southwestern Medical Center, Dallas, TX, USA; 2 SUNY College of Optometry, State University of New York, New York, NY, USA; 3 MassMutual Data Science, Springfield, MA, USA; 4 Retina Foundation of the Southwest, Dallas, TX, USA

**Keywords:** ROP, retinopathy of prematurity, avastin, intravitreal bevacizumab, laser photocoagulation, refractive error development, visual acuity, anisometropia

## Abstract

**Purpose:**

To compare the patterns of longitudinal refractive error development during the first 3.5 years in children with severe retinopathy of prematurity (ROP) treated with intravitreal bevacizumab (IVB) or laser photocoagulation.

**Methods:**

This prospective cohort study enrolled extremely preterm infants (birth weight < 1000 g, gestational age 23–27 weeks) with type 1 ROP from multiple hospitals in Dallas between 1999 and 2017; IVB group (N = 22); laser group (N = 26). Cycloplegic retinoscopy was conducted from 0.04 years corrected age and every 0.5 to 1.0 years thereafter until 3.5 years old. Right eye spherical equivalent (SEQ) and astigmatism, anisometropia, and better-eye visual acuity were analyzed over time.

**Results:**

In all children, both eyes were treated with the same modality. At the final visit, the prevalence of myopia (SEQ ≤ −1D) was 82.7% in the laser group and 47.7% in the IVB group (*P* < 0.05) with a mean SEQ of −8.0D ± 5.8D in the laser group versus −2.3D ± 4.2D in the IVB group (*P* < 0.001). Longitudinal SEQ were best fit with a bilinear model. Before one year, the rate of SEQ change was −5.0D/year in the laser group, but only −3.5D/year in the IVB group (T = −5.14, *P* < 0.001); after one year, there was a significant flattening of these slopes (T = 6.23, *P* < 0.001). Anisometropia in the IVB group was significantly less than in the laser group (*P* < 0.05). Final visual acuity in both groups was similar at 0.47 logMAR (∼ 20/60).

**Conclusions:**

Children with severe ROP treated with IVB developed less myopic refractive error than those treated with laser largely because of a slower rate of refractive change during the first year of life.

**Translational Relevance:**

These findings may inform decisions regarding ROP treatment timing and modality.

## Introduction

Aberrations of refractive development represent significant sequelae of retinopathy of prematurity (ROP).^1^^,^^2^ For example, the Early Treatment of Retinopathy of Prematurity study found that at four years of age, the prevalence of myopia in individuals with severe zone 1 ROP treated with laser photocoagulation was 75.2%, and the prevalence of high myopia (defined as −5.00D or more^3^) was 47.6%.^2^ These rates contrast starkly with estimates of the prevalence of myopia and high myopia of 33% and 4%, respectively, in the general population.^3^^,^^4^ The increased prevalence of high myopia after laser photocoagulation is especially concerning because uncorrected refractive error greater than 5D can lead to significant visual impairment.^3^ High myopia and other refractive aberrations such as astigmatism and anisometropia may also predispose to amblyopia.^5^

A number of studies have compared the effect of alternative treatments for type I ROP on the development of myopia and high myopia. Most studies, including the BEAT-ROP trial, have demonstrated a reduced prevalence of myopia and high myopia among IVB-treated infants compared with laser-treated infants with type I ROP.^6^^–^^11^ Gunay et al. reported a significantly higher prevalence of high myopia in infants with zone 1 ROP vs zone 2 disease and an increase prevalence of high myopia in those treated with laser photocoagulation compared to the IVB treated group.^12^ None of the previous studies examined the pattern of longitudinal refractive error development among infants with type 1 ROP treated with IVB. These previous studies also have not put forth a predictable model of longitudinal refractive development in infants with severe ROP treated with IVB or laser that may be used to facilitate the management of progressive high myopia in this at risk population.^13^

Our previous study found a bilinear pattern or myopia progression in patients treated with laser, with rapid progression of myopia before 1 year with subsequent stabilization.^12^ Observing literature reports of decreased myopia development following treatment with IVB instead of laser, we hypothesized that the longitudinal pattern of refractive development in those treated with IVB would differ from the pattern of those treated with laser photocoagulation. The aims of this study were (1) to evaluate the rate of change and long-term refractive outcome for a prospective cohort with Type I ROP treated with intravitreal bevacizumab (IVB); (2) to compare the rate of change and long-term refractive outcomes of IVB-treated infants with a laser-treated cohort of similar gestational age (GA); (3) to evaluate longitudinal astigmatism, anisometropia and visual acuity changes in these two groups.

## Methods

### Subject Recruitment

Enrollment of subjects for this study occurred at the Retina Foundation of the Southwest under a protocol for prospective evaluation of interventions and outcomes in retinopathy of prematurity that began in 1999 and is ongoing. Subjects were referred for enrollment by two Dallas ophthalmologists who conduct screening and treatment for ROP in multiple neonatal intensive care units in the Dallas metroplex. The research protocol observed the tenets of the Declaration of Helsinki and was approved by the Institutional Review Board of the University of Texas Southwestern Medical Center. Informed consent was obtained from each subject's parent or guardian. Policies and procedures conformed to the requirements of the United States Health Insurance Portability and Accountability Act (HIPAA).

Inclusion criteria: Preterm infants with birth weight less than 1000g, born at 22 to 27 weeks GA, and treated with IVB or laser panretinal photocoagulation for type I ROP (stage 3+ or aggressive posterior ROP in zone 1 or posterior zone 2) were eligible for the study. Exclusion criteria: Infants with retinal detachment (stage 4 or 5 ROP) or concomitant glaucoma were excluded.

Participants were classified into two groups according to treatment. Treatment was determined by the referring physician according to clinical indication. In the IVB group, all infants were treated with a single injection of bevacizumab (0.625mg in 0.025mL). In the laser group, all infants underwent pan-retinal photocoagulation. For secondary analyses, each group was further sub-categorized according to ROP severity (zone 1 and zone 2 disease).

### Measurement of Refractive Error and Visual Acuity

Cycloplegic retinoscopy (1% cyclopentolate) was performed by the referring pediatric ophthalmologist as part of prescribed follow-up care. Only follow up visits where refractive data were collected were included in the analysis. Refraction data were recorded in conventional form as sphere, plus cylinder (CYL), and axis. Initial refraction was conducted between 2 weeks corrected age to 10 months (mean at 4 months) and every 6-12 months thereafter. In cases where study participants had collected data beyond 3.5 years, we included only data from before 3.5 years old. Each patient had at least 3 cycloplegic refractions performed. Visual acuity was measured with Teller cards^14^ (reported in cycle per degree) and converted into logMAR.

### Data Analysis and Statistics

Using a custom spreadsheet (Excel; Microsoft, Inc., Redmond, WA), refractive errors were converted into their power vector components: spherical equivalent (SEQ = sphere + 0.5*CYL), J_0_ (positive J_0_ indicates with-the-rule astigmatism), negative J_0_ (indicates against-the-rule astigmatism), and J_45_ (oblique astigmatism: positive J_45_ indicates 135° astigmatism whereas negative J_45_ indicates 45° astigmatism).^15^ Because of high intereye correlation for refractive data (SEQ *R* = 0.89, *T* = 28.2, *P* < 0.001), we analyzed only right eye data, except with evaluation of anisometropia. Because visual acuity may be impacted by amblyopia, we report data for the better-seeing eye.

Myopia was defined as SEQ ≤ −1.00D and high myopia as SEQ ≤ −5.00D. Anisometropia was calculated by taking the absolute value of the difference in SEQ between the right eye and the left eye. Significant anisometropia was defined as a interocular SEQ difference ≥ 1D. Corrected age was used for all analyses: Corrected Age = Postnatal Age – [40-GA at birth]. Unless otherwise specified, hereafter we use the term “age” to refer to corrected age.

Data analysis was performed and plotted using R 3.5.0 Statistics. (R Core Team. URL: http://www.R-project.org/). Descriptive statistics are presented as mean ± standard deviation (SD). Analysis of variance tests were applied to compare initial and final mean SEQ results between treatment groups and across zones. Comparisons of the prevalence were conducted by calculating confidence intervals with a *P* value = 0.05.

### Longitudinal Models of SEQ, Astigmatism, Anisometropia and Visual Acuity

To estimate the rate of individual SEQ change with age, a linear mixed effect model was used. The mixed effect model uses longitudinal information from each individual and provides comparisons between treatment groups, as well as comparisons within each group according to zone. Treatment modality was classified as a fixed effect and the individual as a random effect.

Using the iterative weighted least square method, refraction data for SEQ were fit with a bilinear model. The bilinear model was used to describe two linear relations between refractive error and age, one for ages less than the transition point and one for ages beyond the transition point. The Akaike Information Criterion (AIC),^16^^,^^17^ a widely used method for model selection that considers the model complexity and goodness of fit of the model to the data, was used here to optimize the selection of the transition point with the following procedure: (1) set the search interval as [0.3, 2] with a step of 0.1; (2) test each step uniformly distributed locations by fitting the model and calculating the AIC value; (3) find the transition point corresponding to the smallest AIC value; (4) finish the model with this transition point.

In any case where we observed no significant difference (by *t*-test of the mixed effect model) between the slopes of the two lines in the bilinear fit, data were reanalyzed using a simple linear model fit by the iterative weighted least square method. We conducted similar analyses on anisometropia. Astigmatism (J_0_, J_45_, magnitude of astigmatism CYL) and visual acuity data were fit with a linear model.

## Results

### Baseline Characteristics

**Figure 1.**
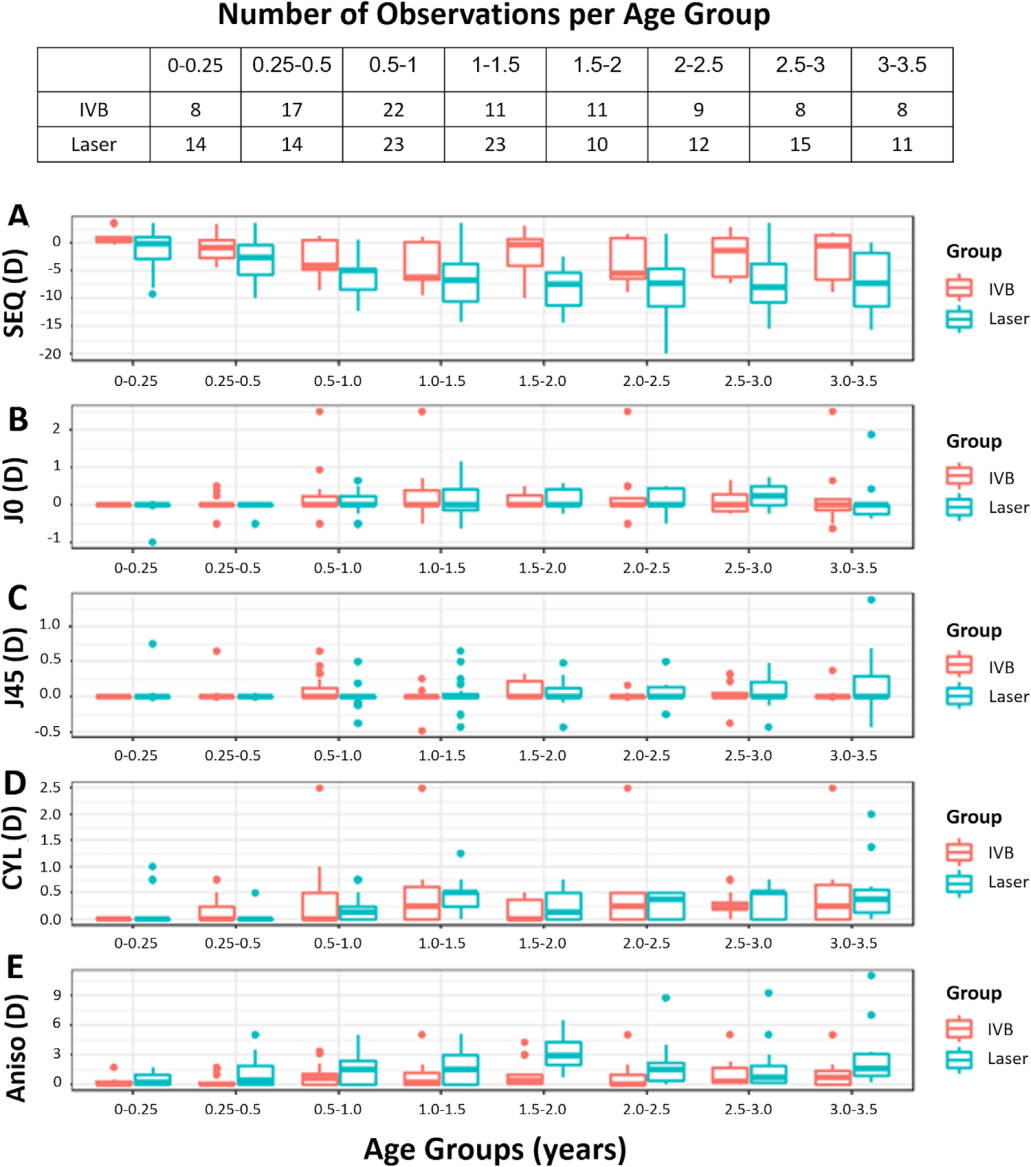
Boxplot for age groups. (A) SEQ, (B) J0, (C) J45, (D) Cylinder, and (E) Anisometropia.

Three IVB-treated infants and four laser-treated infants were excluded because of retinal detachment (two with bilateral total retinal detachment and one with bilateral partial detachment involving the macula). One infant originally recruited from the IVB group, was later diagnosed with familial exudative vitreoretinopathy and excluded. Following these exclusions, a total of 48 preterm infants were enrolled with 22 in the IVB group and 26 in the laser group. The Table presents baseline characteristics. The two groups were similar in prevalence of zone 1 and zone 2 disease, all had plus disease, the percentage of stage 3, and the length of follow-up. In all cases, infants received the same treatment in each eye. On average, IVB treatment was administered 1.5 weeks earlier than laser treatment. Figure 1 includes information about the number of participants who completed refraction visits at each six-month interval.

**Table.** Summary of Demographic and Clinical Features of the IVB and Laser Groups

	IVB Group	Laser Group	*P* Value
Total Patients N	22	26	
Female N (%)	7 (32%)	13 (50%)	0.32
Gestational age at birth(week), mean ± SD (min to max)	24.5 ± 1.3 (23–27)	24.7 ± 1.2 (23–27)	0.54
Birth weight (g), mean ± SD (min to max)	686.4 ± 144.2 (475, 992)	597.5 ± 130.4 (397, 879)	0.03^*^
Stage of ROP in OD/OS	2+: 6/6 3+: 16/16	2+: 7/8 3+: 19/18	
Zone 1	8 (36%)	9 (35%)	
Zone 2	14 (64%)	17 (65%)	
ROP treatment timing (week, mean ± SD (min to max))	34.4 ± 1.3^†^ (33–37)	35.9 ± 2.2 (32–39.4)	0.005^*^
Number of refraction visits, median (min; max)	4 (3; 7)	5 (3; 7)	0.23
Age of first refraction (YR) mean ± SD (min to max)	0.32 ± 0.2 (0.02, 0.83)	0.25 ± 0.2 (0, 0.73)	0.19
Age of final refraction (YR) mean ± SD (min to max)	2.7 ± 0.5 (1.8, 3.5)	2.8 ± 0.5 (2.0, 3.5)	0.30
Length of follow up (YR) mean ± SD (min to max)	2.4 ± 0.4 (1.8, 2.8)	2.6 ± 0.3 (2.0, 3.0)	0.03^*^

* •••

† Five of them had laser treatment later. In three cases this was for a late focal peripheral detachment repair (at 46–80 weeks GA) and in two cases for recurrence at 39 and 53 weeks GA. Laser treatment for these individuals was performed at 80.7, 38.9, 49.7, 46.7, and 53.3 weeks.

### SEQ and Myopia

Figure 1A contains box plots representing of the range of SEQ values over consecutive time points in the study. Refractive error was similar between the two groups at the initial visit (*F* = 2.0, *df* = 1, *P* = 0.164). At the final visit, SEQ for the laser group was on average −8.00D ± 5.84D (−20.00D to +3.50D), whereas SEQ for the IVB group was −2.38D ± 4.18D (−10.00D to +2.75D). Additional details regarding the initial and final refractive error of both groups can be found in Appendix 1. There was a significantly more myopic shift in the laser group than in the IVB group (*F* = 14.2, *df* = 1,*P* < 0.001) during the study**.**

**Figure 2.**
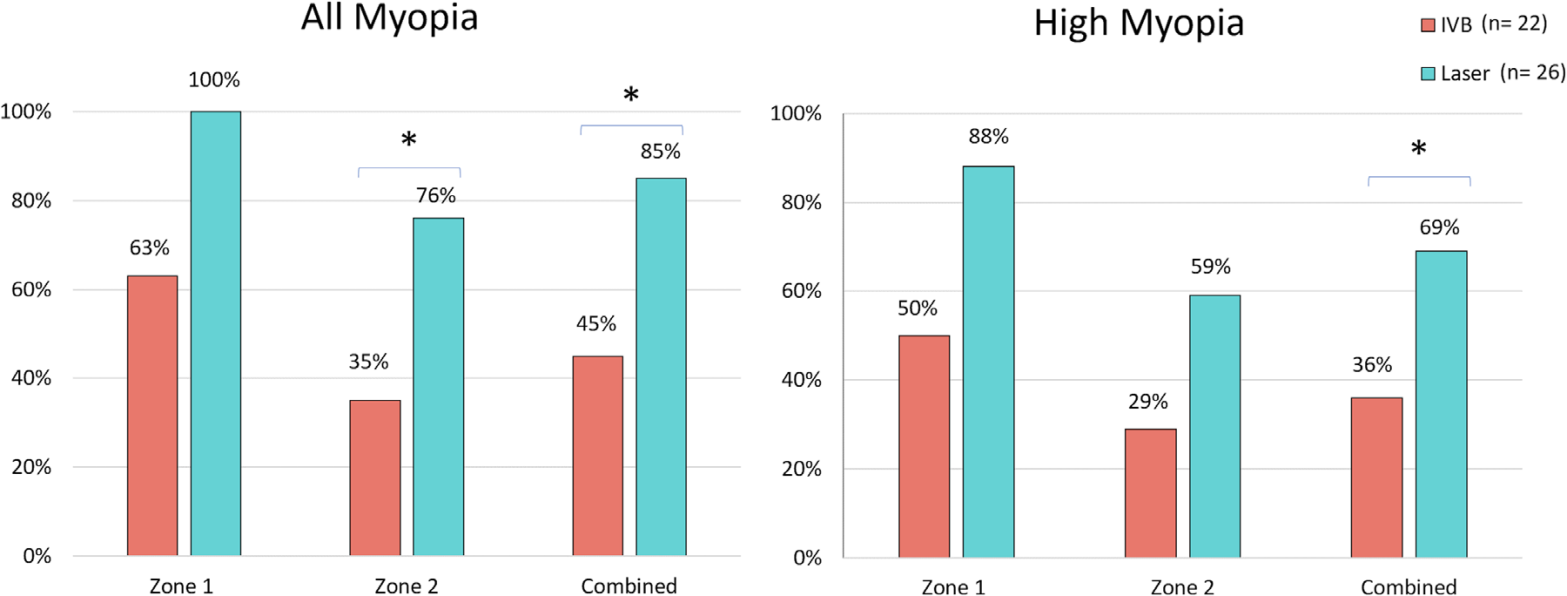
Bar graph displaying the final prevalence of myopia and high myopia by treatment (combined) and according to ROP severity (zone 1 and zone 2). The prevalence of myopia and high myopia was uniformly higher in infants treated with laser. These differences reached statistical significance (*P* < 0.05) in the combined groups and in the zone 2 comparison of any myopia. (Myopia is defined as SEQ −1D or more; high myopia is defined as SEQ −5D or more). *Asterisk* indicates *P* < 0.05.

Figure 2 displays the prevalence of myopia and high myopia in zone 1 and 2 by treatment group. Overall, combining zone 1 and 2, there was significant difference in the prevalence of myopia (Laser: 85%, IVB: 45%, odds ratio [OR] = 6.6; 95% confidence interval [CI]: 1.7–25.6, *P* = 0.004) and high myopia (Laser: 69%, IVB: 36%, OR = 6.9; 95% CI: 1.2–13.1, *P* = 0.02) between the two treatment groups. In addition, the mean SEQ of study participants from both groups with zone 1 disease was significantly more myopic than that of participants with zone 2 disease. Study participants with Zone 1 ROP had statistically more myopic mean SEQ (zone 1: SEQ = −5.4D ± 5.8D; zone 2: SEQ = −3.1D ± 4.4D; *F* = 4.4, *df* = 1, *P* = 0.04) and significantly higher prevalence of myopia compared with eyes with zone 2 ROP at the last visit.

### Longitudinal Model of SEQ

**Figure 3.**
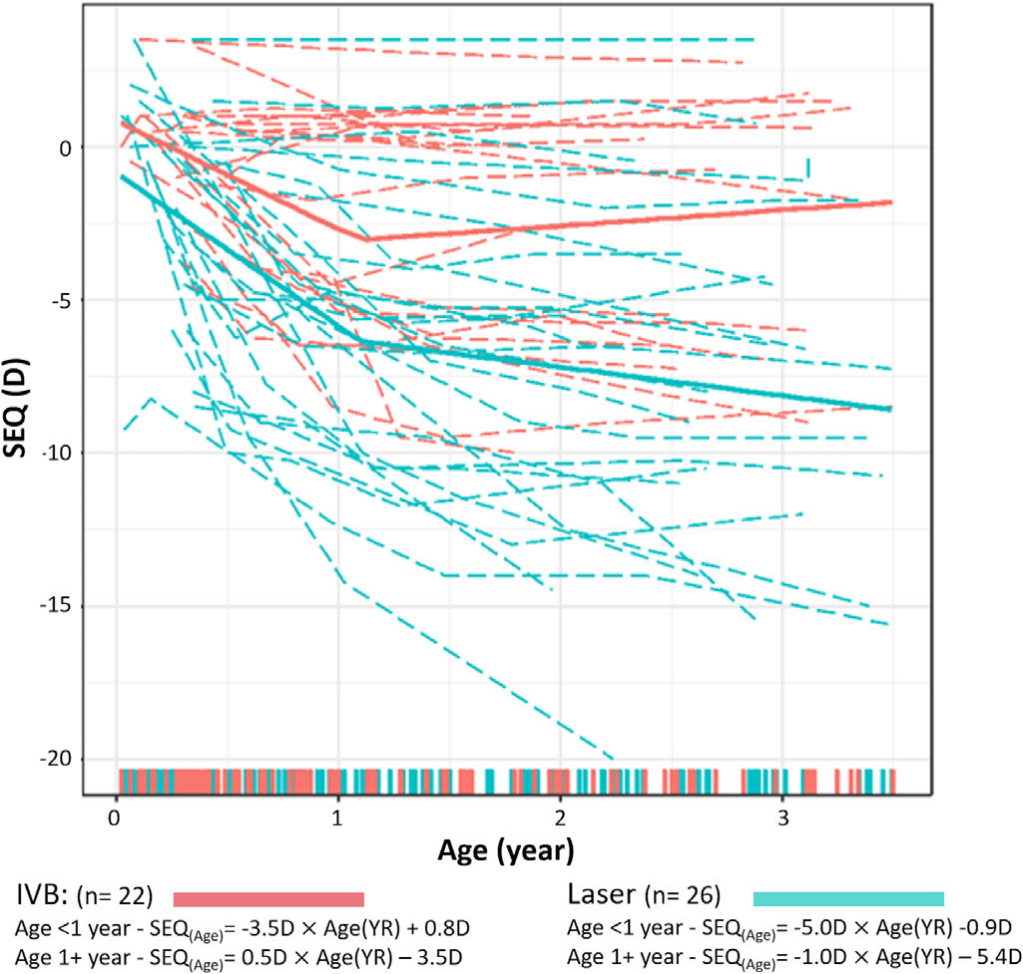
Individual development of SEQ and the best-fit models over the first 3.5 years of corrected age for the IVB and laser groups. The *red color* indicates the IVB group; the *cyan color* indicates the laser group. The *dashed* *lines* indicate individual longitudinal data; the *solid line* indicates the best-fit bilinear model for each treatment group. The formulas for each line are given below the model. The *shaded areas* indicate 95% CI. The *colored bars* along the x-axis represent the time points for individual measurements from each group.

Figure 3 displays the longitudinal data from each individual and the best-fit model for longitudinal change of SEQ for the IVB and laser groups. Our approach produced multiple models with possible transition points from 0.3 to 2.0 years old, each with an AIC value reflecting the goodness of fit of that model for the study data. AIC values ranged from 1044 to 1061. The model with the smallest AIC was a bilinear model with a transition point at 1.1 years old. Based on this model, longitudinal SEQ changes in the IVB group were fit by the following two equations:

Corrected age <1.1 YR:

$$\begin{array}{c} \mathrm{SE}Q_{\left(\mathrm{Age}\right)}=-3.5D\times \mathrm{Age}\left(\mathrm{YR}\right)+0.8D \end{array}$$

Corrected age 1.1 to 3.5 years:

$$\begin{array}{c} \mathrm{SE}Q_{\left(\mathrm{Age}\right)}=0.5D\times \mathrm{Age}\left(\mathrm{YR}\right)-3.5D \end{array}$$

SEQ changes in the laser group were fit by the following two equations:

Corrected age <1.1 YR:

$$\begin{array}{c} \mathrm{SE}Q_{\left(\mathrm{Age}\right)}=-5.0D\times \mathrm{Age}\left(\mathrm{YR}\right)-0.9D \end{array}$$

Corrected age 1.1 to 3.5 years:

$$\begin{array}{c} \mathrm{SE}Q_{\left(\mathrm{Age}\right)}=-1.0D\times \mathrm{Age}\left(\mathrm{YR}\right)-5.4D \end{array}$$

During the first 1.1 years, the SEQ significantly decreased over time at an average rate of −3.5D/YR in the IVB group (*T* = −6.7, *df* = 165, *P* < 0.001), the rate of SEQ change in the laser group was significantly faster at −5.0D/YR (*T* = −5.14, *df* = 165, *P* < 0.001). After 1.1 years, the rate of change became slower for both groups (*T* = 6.23, *df* = 165, *P* < 0.001).

### Longitudinal Models of Cylinder, Anisometropia, and Visual Acuity (Figs. 1C–1E)

#### Magnitude of Astigmatism

We analyzed J_0_ and J_45_ of astigmatism over 3.5 years and found no significant difference between the IVB and laser groups (*T* = −0.50, *df* = 46, *P* = 0.61 for J0; *T* = −0.18, *df* = 46, *P* = 0.86 for J45). We therefore modeled only the magnitude of astigmatism (CYL). At the final visit, CYL for the laser group was on average 0.46D ± 0.45D (0.00D to 2.00D), whereas CYL for the IVB group was 0.36D ± 0.55D (0.00D to +2.50D). There was no significantly different CYL in two groups (*t* = −0.67, *df* = 40.4, *P* = 0.50) during the study**.** At the final visit, 16/26 (61.5%) in the laser group and 9/22 (41%) in the IVB group had significant astigmatism (CYL ≥ 1D), but these differences did not reach statistical significance (OR = 2.3; 95% CI, 0.7–7.4; *P* = 0.15).

Longitudinal cylinder changes were fit by the equation: CYL(Age) = 0.2D × Age (YR) + 0.3D. There was a significant increase in cylinder magnitude with age (*T* = 5.49, *df* = 167, *P* < 0.001), but there were no significant differences between the two treatment groups (*T* = −0.17, *df* = 46, *P* = 0.86).

### Anisometropia

At the final visit, 16 of 26 (61.5%) in the laser group and 8 of 22 (36%) in the IVB group had significant anisometropia (interocular SEQ difference ≥ 1D), but these differences in prevalence did not reach statistical significance (OR = 2.8; 95% CI, 0.9–3.2; *P* = 0.08). At the final visit, anisometropia for the laser group was on average 2.14D ± 2.50D (0.00D to 11.00D), whereas anisometropia for the IVB group was 0.94D ± 1.48D (0.00D to +5.00D). There was significant higher anisometropia in the laser group than in the IVB (*t* = 2.06, *df* = 41.6, *P* = 0.45) during the study**.** The overall magnitude of significant anisometropia in the laser group was significantly higher than in the IVB group (*T* = 2.5, *df* = 46, *P* < 0.05).

**Figure 4.**
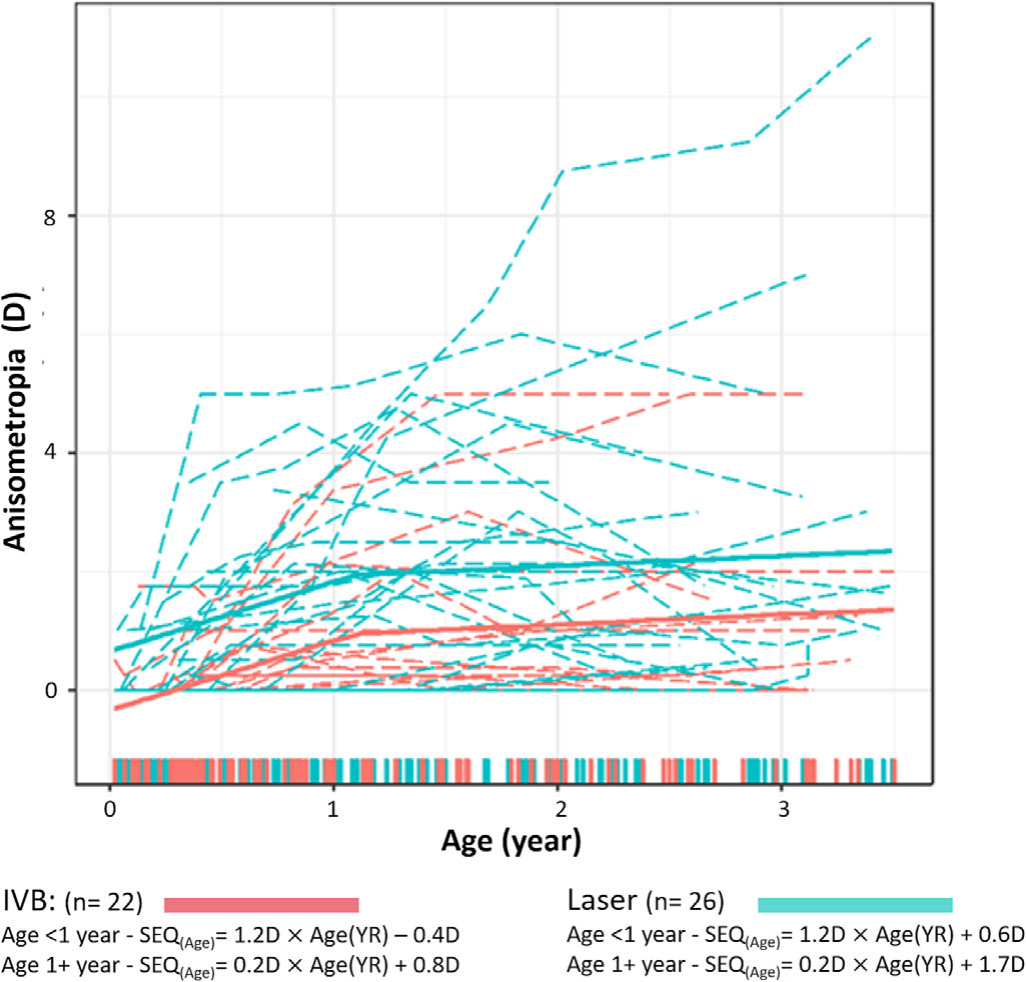
Individual development of anisometropia and the best-fit models over the first 3.5 years of corrected age for the IVB and laser groups. The *red color* indicates the IVB group; the *cyan color* indicates the laser group. The *dashed* *lines* indicate individual longitudinal data; the *solid line* indicates the best-fit bilinear model for each treatment group. The formulas for each line are given below the model. The *shaded areas* indicate 95% CI. The *colored bars* along the x-axis represent the time points for individual measurements from each group.

The pattern of anisometropia change over time for both treatment groups followed a bilinear pattern (Fig. 4). Irrespective of treatment modality, the transition point for the best-fit model occurred at 1.1 year corrected age. Longitudinal anisometropia changes in the IVB group were fit by the following two equations:

Corrected age <1.1 YR:

$$\begin{array}{c} \mathrm{Anisometropi}a_{\left(\mathrm{Age}\right)}=1.2D\times \mathrm{Age}\left(\mathrm{YR}\right)-0.4D \end{array}$$

Corrected age 1.1 to 3.5 years:

$$\begin{array}{c} \mathrm{Anisometropi}a_{\left(\mathrm{Age}\right)}=0.2D\times \mathrm{Age}\left(\mathrm{YR}\right)+0.8D \end{array}$$

SEQ changes in the laser group were fit by the following two equations:

Corrected age <1.1YR:

$$\begin{array}{c} \mathrm{Anisometropi}a_{\left(\mathrm{Age}\right)}=1.2D\times \mathrm{Age}\left(\mathrm{YR}\right)+0.6D \end{array}$$

Corrected age 1.1 to 3.5 years:

$$\begin{array}{c} \mathrm{Anisometropi}a_{\left(\mathrm{Age}\right)}=0.2D\times \mathrm{Age}\left(\mathrm{YR}\right)+1.7D \end{array}$$

Before 1.1 years of age, there was a significant increase of anisometropia (1.2D/year) in the two groups (*T* = 4.55, *df* = 166, *P* < 0.001). After 1.1 years, the slopes were significantly slower (*T* = −2.9, *df* = 166, *P* < 0.01).

### Visual Acuity

Longitudinal visual acuity (VA) of the better-seeing eye for both groups were fit by the following equation:

$$\begin{array}{c} VA_{\left(\mathrm{Age}\right)}=-0.15\mathrm{logMAR}\times \mathrm{Age}\left(\mathrm{YR}\right)+0.9\mathrm{logMAR}. \end{array}$$

The slope of visual acuity was significant improved over age (*T* = −7.6, *df* = 82, *P* < 0.001). There were no significant differences between the two groups (*T* = 0.12, *df* = 44, *P* = 0.91). By 3.5 years, the average VA in both groups was 0.5 logMAR, (20/60 Snellen equivalent).

## Discussion

In this study we compared longitudinal refractive outcomes between two prospective cohorts of extremely preterm infants with Type I ROP after treatment with either IVB or laser. Our work is noteworthy for the length of prospective follow-up of both refractive error and visual function in these two treatment groups. In fact, many of our patients were refracted before 12 months of age. Refractive change in both groups were best fit with a bilinear pattern, and we identified, using the AIC, a distinct transition point in both groups at 1.1 years. Children with severe ROP who were treated with IVB developed less myopic refractive error than those treated with laser largely because of a slower rate of refractive change during the first year of life. Afterward, the rate of myopia progression slowed significantly in both groups. Consequently, treatment with IVB was associated with a significantly lower prevalence of high myopia at 3.5 years old. Our results are consistent with our previously published findings for laser-treated infants,^12^ and confirm our hypothesis that the pattern of refractive error development differs between infants treated with IVB and those treated with laser. The rapid increase in myopia during the first year of life suggests that early monitoring and timely optical correction may be indicated to improve long-term visual outcomes.

**Figure 5.**
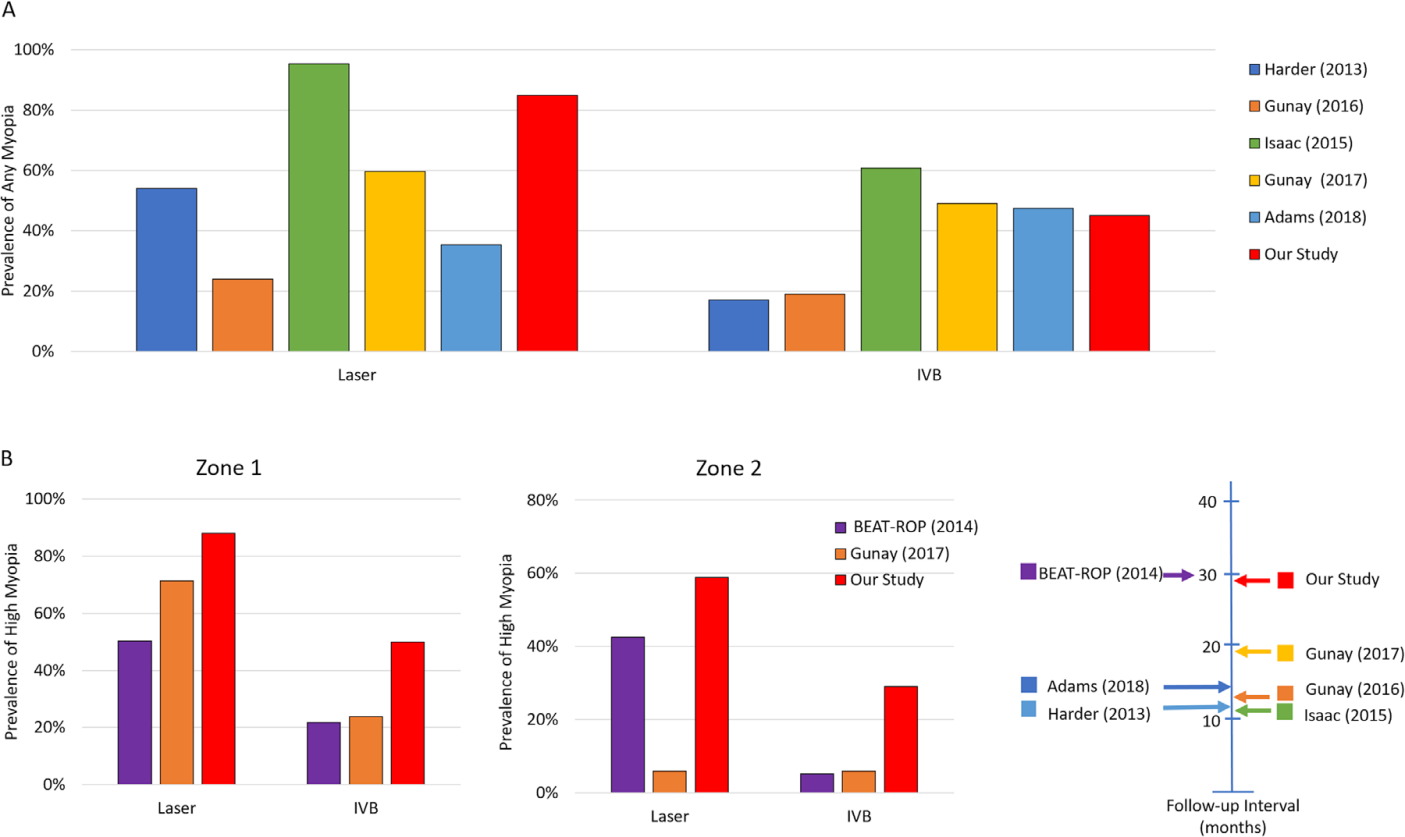
Comparison of the prevalence of myopia (A) and high myopia (B) from this study and the prevalence in populations of comparable gestational age and birth weight described by other groups in the literature.

Figure 5 shows a comparison of our refractive error results compared to other published studies that evaluated the prevalence of myopia and high myopia in IVB- and laser-treated infants. Our findings are consistent with those of the BEAT-ROP trial. They are also in agreement with Harder et al.^9^ and other groups who have published similar retrospective studies that demonstrated a definite correlation between IVB therapy and a reduced prevalence of myopia.^6^^–^^8^^,^^10^ Overall, in both zone 1 and 2 disease (Fig. 5B), IVB therapy was associated with a lower prevalence of myopia and high myopia compared with laser treatment.

The odds of development of significant astigmatism were higher in the laser cohort in this study than in the IVB cohort, but the differences did not reach statistical significance. Previous studies have reported astigmatism in Type I ROP with ranges of cylinder power from 0.6D ± 0.8D to 1.3D ± 0.9D in IVB treatment and 1.4D ± 0.7D to 2.1D ± 1.1D in laser treatment.^9^^,^^14^^,^^15^ With one exception,^9^ none of these studies found a significant statistical difference in CYL between laser and IVB-treated subjects. Notably, the magnitude of astigmatism CYL was observed in this study to increase with age in both groups, which is consistent with our previous findings.^12^

The development of anisometropia among participants in this study also followed a bilinear pattern with the greatest increases occurring in both groups before a transition point, occurring at 1.1 years. Furthermore, we found significantly less anisometropia in the IVB group than in the laser group. The prevalence of anisometropia in this study was 61.5% in the laser group and 36% in the IVB group. Although these differences in prevalence did not reach statistical significance, their trend is in agreement with the observations of Gunay et al.,^18^ who noted a significantly higher prevalence of anisometropia in children with ROP treated with laser (66.7%) compared with those treated with IVB (20%, *P* = 0.009). Our results for the IVB cohort are also in agreement with the overall prevalence in IVB-treated infants reported by Chen et al.^19^ of 35%.^13^ Anisometropia may occur more frequently in laser-treated infants because asymmetric disease may necessitate the need for a greater treatment area in one eye compared to the other. In the BEAT-ROP study, myopia increased with more laser applications, (−0.14 D for every 100 laser applications given).^6^ An added benefit of IVB treatment compared to laser may be a reduced risk of anisometropia and amblyopia.

The visual acuity of the better-seeing eye in both the laser and IVB cohorts in this study improved over time along a similar trajectory. By 3.5 years, both groups had an average visual acuity of approximately 20/60, which is within the normal range for healthy children of similar age.^20^^,^^21^ Visual acuity is not only related to optics but also related to visual cortex level function.

### Contributions, Implications, Limitations

Our work is noteworthy for the length of prospective follow up of both refractive error and visual function in our cohort of extremely premature infants with advanced ROP. One unique aspect of this study is the collection of refractive data during the first year of life. This permitted us to identify the unique differences between the first year of life and subsequent years in our models. Our models of SEQ, astigmatism, anisometropia, and visual acuity development with the use of the AIC for identification of the transition points in development also constitute a unique and valuable contribution.

Myopia associated with ROP relates to abnormalities of the anterior segment.^7^^,^^22^^–^^31^ Fielder and Quinn^32^ suggested myopia associated with ROP may develop because damaged peripheral retina physically constrains the development of the anterior segment. In support of this mechanical restriction hypothesis, they observed that eyes treated with laser developed less myopia than eyes treated with cryotherapy and suggested this is because laser is less tissue-destructive. It may be that the longitudinal patterns of refractive error change in the subjects of our study reflect the preservation of more retinal tissue in IVB-treated eyes compared to those treated with laser. Whether the ultimate changes occur because of physical restraint of scarring, modification of growth signals, or a separate etiology altogether is unclear.

The parallel trajectories observed in our models of longitudinal refractive error development in both IVB- and laser-treated infants with stabilization at 1.1 years suggest that the reasons for bilinear growth and stabilization after the transition point may be independent of treatment. The normal timeline of ocular development in healthy individuals involves dramatic changes in axial length, ocular volume, and other parameters in the first six to 48 months.^33^ Perhaps the eye is more susceptible to interference during these initial months of rapid growth than afterward. These findings suggest that refractive outcomes in infants with ROP might be optimized by adjusting the timing of interventions.

In practice, many different factors may influence a physician's decision to choose IVB therapy over laser.^34^ The possibility of a more favorable refractive outcome including reduced myopia and anisometropia may provide an added benefit. IVB therapy, however, may result in persistent avascular retina and possible late reactivation of ROP. In our study, five IVB-treated infants received delayed laser treatment at a mean of 53.9 weeks for either focal detachment or ROP recurrence. The prevailing hypothesis is that peripheral retina influences lens development, and high myopia in ROP is mostly due to increased lens power. The age at which laser is applied could be a factor in developing myopia; that is, early laser treatment may disrupt anterior segment development whereas later laser treatment does not. Moreover, the extent of laser treatment has been shown to influence the degree of myopia. When laser is used as primary treatment for Type I ROP, there is a substantial destruction of the retina, which is likely to cause more disruption of lens development compared with the less aggressive laser treatment at 50 to 60 weeks for persistent avascular retina or focal peripheral detachment.

In a dose de-escalation trial of IVB for infants with type 1 or type 2 ROP, Crouch et al.^35^ found at 12 months that 31% of treated eyes were not Zone III or fully vascularized and 27 study eyes required laser treatment for persistent avascular retina or recurrent ROP. If the pattern of stabilization in refractive change observed in our study were reflective of ocular growth patterns, it is possible that secondary laser treatment after IVB would have less of an effect on refractive error. Indeed, Crouch et al.^35^ reported that the mean SEQ was similar between infants treated with subsequent laser and those who required no laser treatment after initial IVB therapy. The reasons why there was no myopic shift in the laser treatment group are unclear. Perhaps fewer laser spots were applied in these cases, because infants of smaller avascular zones in these infants than in infants receiving primary laser therapy. Additionally, providers treating persistent avascular retinas may have approached secondary laser treatment less aggressively than they would have approached primary laser therapy.

There are several limitations to this study. (1) All patients in this study were from the Dallas area, so our findings may not be directly applicable to other populations. (2) We did not evaluate other clinical considerations relevant to the decision to treat with IVB or laser such as efficacy,^36^ the possibility of long-term ocular or systemic adverse effects of IVB, or the need for and timing of retreatment.^36^ (3) Our study evaluated only the standard dosage of 0.625mg IVB even though lower dosages of IVB may also be efficacious.^37^^,^^38^ (4) Our study evaluated only refractive error. Other biometric measures would be very helpful in understanding the pathophysiology of myopia associated with ROP (Lenis et al.^30^); however, taking these measurements in young infants is difficult and was beyond the scope of this study. (5) The treatment decisions for patients included in this study were not random, which may have introduced a selection bias. Although our study populations were comparable in GA, birth weight, disease severity and length of follow-up, there may have been other distinguishing clinical features in these infants that influenced the physicians’ treatment choices, timing and response to treatment or and predilection for myopia. Patients in this study were also recruited over a lengthy period of time that spanned the introduction of IVB into mainstream ROP clinical practice. (6) Finally, this study was limited by small sample size. This may have limited our ability to detect significant differences between groups. For example, although the odds ratios for the development of astigmatism and anisometropia in the laser group versus the IVB group >2, these differences did not reach significance.

## Conclusions

The prevalence of myopia was lower in preterm children who were treated with IVB for severe ROP than it was in children treated with laser. A critical period of rapid change of SEQ that occurs in the first year of life accounts for this difference. IVB-treated children also experienced lower anisometropia, but levels of significant astigmatism were similar between the two groups, and visual acuity improved for both groups with age.

## Supplementary Material

Supplement 1
