# Supplementary material for: Longitudinal Development of Refractive Error in Children Treated With Intravitreal Bevacizumab or Laser for Retinopathy of Prematurity
Source: Transl Vis Sci Technol. 2021 Apr 15;10(4):14. doi: 10.1167/tvst.10.4.14 (PMC8054622; doi:10.1167/tvst.10.4.14)
Supplement: Supplement 1 [file tvst-10-4-14_s001.docx]

**Appendix1.** Summary of SEQ of the IVB and laser groups at the initial visits and final visits with Zone information.

|  | Initial visit | | | | Final visit | | | |
| --- | --- | --- | --- | --- | --- | --- | --- | --- |
|  | IVB | | Laser | | IVB | | Laser | |
|  | Zone 1 | Zone 2 | Zone 1 | Zone 2 | Zone 1 | Zone 2 | Zone 1 | Zone 2 |
| SEQ (D) | -1.53±2.35 | 0.18±1.85 | -2.05±4.23 | -1.40±3.07 | -3.66±4.51 | -1.65±3.96 | -10.69±5.73 | -6.58±5.54 |

ANOVA test: Initial vs final, F=25.5, P<0.001*; IVB vs Laser, F=15.56, P<0.001*; Zone 1 vs Zone 2, F value=5.67, P=0.02*.


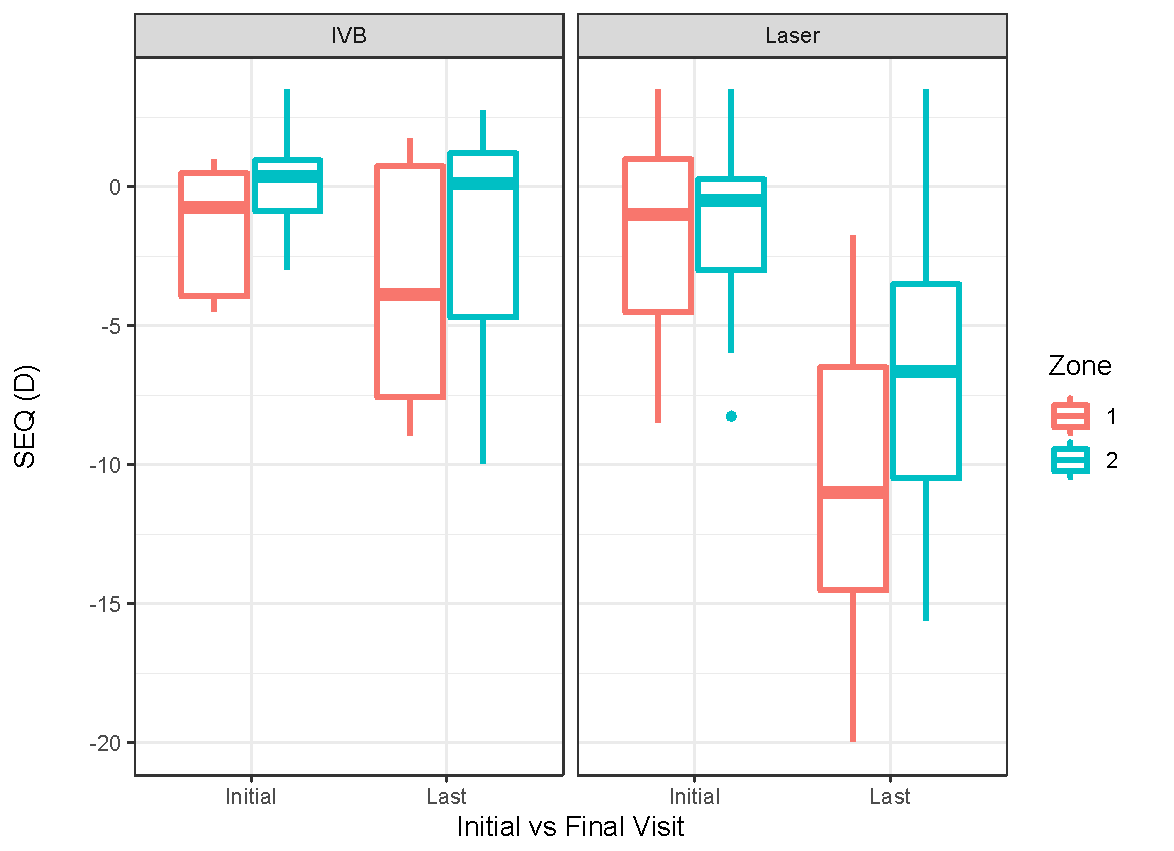


# Appendix 2: Models in a table:

Bilinear model fitting for right eye SEQ

SEQ=intercept + a*age + b*group + c*piece2+ d*age*group

b=0 for IVB group and b=1 for laser group

d=0 for IVB group and d=1 for laser group

Piece2=(age-1.1) when age > 1.1 years

| SEQ |  | Value for parameter | DF | T-value | P-value |
| --- | --- | --- | --- | --- | --- |
| Transition point=1.1y | Intercept | 0.84 | 165 | 0.93 | 0.35 |
|  | a | -3.54 | 165 | -6.99 | <0.001* |
|  | b | -1.71 | 46 | -1.45 | 0.15 |
|  | c | 4.06 | 165 | 6.23 | <0.001* |
|  | d | -1.45 | 165 | -5.14 | <0.001* |

Bilinear model fitting for anisometropia

Anisometropia=intercept +a*age+ b*group (1,2)+ c*piece2

b=0 for IVB group and b=1 for laser group

Piece2=(age-1.1) when age > 1.1 years

| Anisometropia |  | Value | DF | T-value | P-value |
| --- | --- | --- | --- | --- | --- |
| Transition point=1.1y | Intercept | -0.35 | 166 | -1.02 | 0.30 |
|  | a | 1.18 | 166 | 4.55 | <0.001* |
|  | b | 0.99 | 46 | 2.53 | 0.01* |
|  | c | -1.01 | 166 | -2.90 | <0.01* |

Linear model fitting for right eye cylinder

CYL=intercept +a*age

| CYL |  | Value | DF | T-value | P-value |
| --- | --- | --- | --- | --- | --- |
|  | Intercept | 0.32 | 167 | 2.70 | <0.01* |
|  | a | 0.19 | 167 | 5.49 | <0.001* |

Linear model fitting for better-seeing eye visual acuity

Visual Acuity=intercept +a*age

| Visual acuity in better-seeing eye |  | Value | DF | T-value | P-value |
| --- | --- | --- | --- | --- | --- |
|  | Intercept | 0.94 | 82 | 22.96 | <0.001* |
|  | a | -0.15 | 82 | -7.64 | <0.001* |
